# BRCA2‐positive lung adenocarcinoma treated with olaparib: A case report

**DOI:** 10.1002/rcr2.1317

**Published:** 2024-03-07

**Authors:** Takumi Motohashi, Kazutoshi Isobe, Takahiro Yoshizawa, Yusuke Usui, Hiroshige Shimizu, Muneyuki Sekiya, Shion Miyoshi, Yasuhiko Nakamura, Naohisa Urabe, Susumu Sakamoto, Sakae Homma, Sota Sadamoto, Naobumi Tochigi, Kazuma Kishi

**Affiliations:** ^1^ Division of Respiratory Medicine Toho University School of Medicine Tokyo Japan; ^2^ Department of Pathology Toho University School of Medicine Tokyo Japan

**Keywords:** adenocarcinoma, BRCA2 gene mutation, cancer genomic profiling test, olaparib

## Abstract

A 66‐year‐old woman was found to have abnormal shadows on a chest radiograph at a previous hospital 4 years ago, which led to a diagnosis of lung adenocarcinoma, cT2aN1M1b stage IVA. First‐line treatment included carboplatin and paclitaxel plus thoracic radiotherapy and stereotactic radiation therapy for brain metastases. The patient later underwent second‐line pemetrexed treatment, followed by third‐line nivolumab, fourth‐line docetaxel and bevacizumab, fifth‐line tegafur‐gimeracil‐oteracil, and sixth‐line gemcitabine. Two years ago, after observing an increase in the primary lesion and carcinoembryonic antigen levels (104.0 ng/mL), a computed tomography‐guided biopsy was performed from the primary site of lung cancer. A cancer genomic profiling test (FoundationOne® CDx cancer genome profile) revealed a breast cancer susceptibility (BRCA) 2 gene mutation. Therefore, she started taking olaparib. The treatment led to stable disease for approximately 2 years.

## Graphical abstract

A 66‐year‐old woman diagnosed with stage IVA lung adenocarcinoma underwent multiple lines of treatment, including chemotherapy and targeted therapies. After discovering a BRCA2 gene mutation through genomic profiling, she initiated olaparib treatment, resulting in approximately 2 years of stable disease.

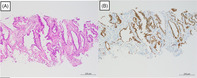

## INTRODUCTION

The breast cancer susceptibility (BRCA)2 gene is the germ cell lineage that produces a protein necessary for repairing double‐stranded DNA. The targeted drug for this mutation is polyadenosine diphosphate‐ribose polymerase (PARP) inhibitors, but currently, its approved indications are limited to breast cancer, prostate cancer, ovarian cancer, and pancreatic cancer worldwide. This is a case report of olaparib use for BRCA2‐positive primary lung cancer.

## CASE REPORT

A 66‐year‐old woman presented with abnormal shadows on a chest radiograph and was diagnosed with lung adenocarcinoma cT2aN1M0 stage IIIB at a previous hospital 4 years ago. She was referred to our hospital for treatment of lung cancer. Around that time, a mass was detected in her right breast and a needle biopsy confirmed apocrine adenocarcinoma. However, after consulting with a breast cancer specialist, treatment for lung cancer was prioritized. Initial lung cancer treatment consisted of concurrent chemoradiotherapy with carboplatin and paclitaxel plus thoracic radiotherapy (60 Gy), but subsequent brain magnetic resonance imaging (MRI) revealed an increase in brain metastasis. Stereotactic brain radiation therapy (18 Gy) was administered. She had a smoking history of 15 pack‐years, and no exposure to asbestos. Physical examination results were unremarkable. The blood test results showed an elevated serum carcinoembryonic antigen (CEA) level of 104.0 ng/mL.

**FIGURE 1:**
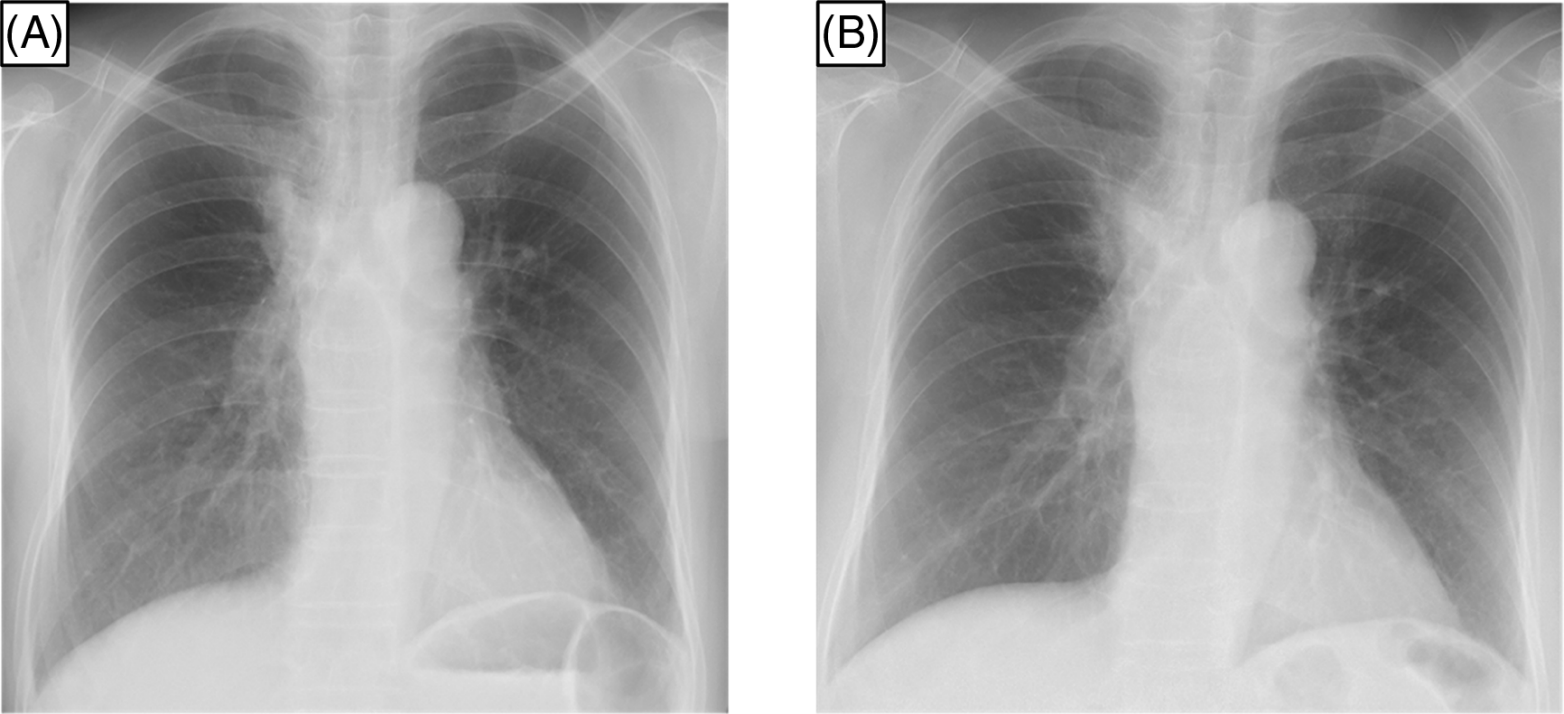
Results of chest radiography imaging. (A) Chest radiograph before starting the sixth treatment, revealing infiltrative shadows in the right upper lung field. (B) Chest radiograph at the time of admission, depicting an increase in the size of the infiltrative shadows.

**FIGURE 2:**
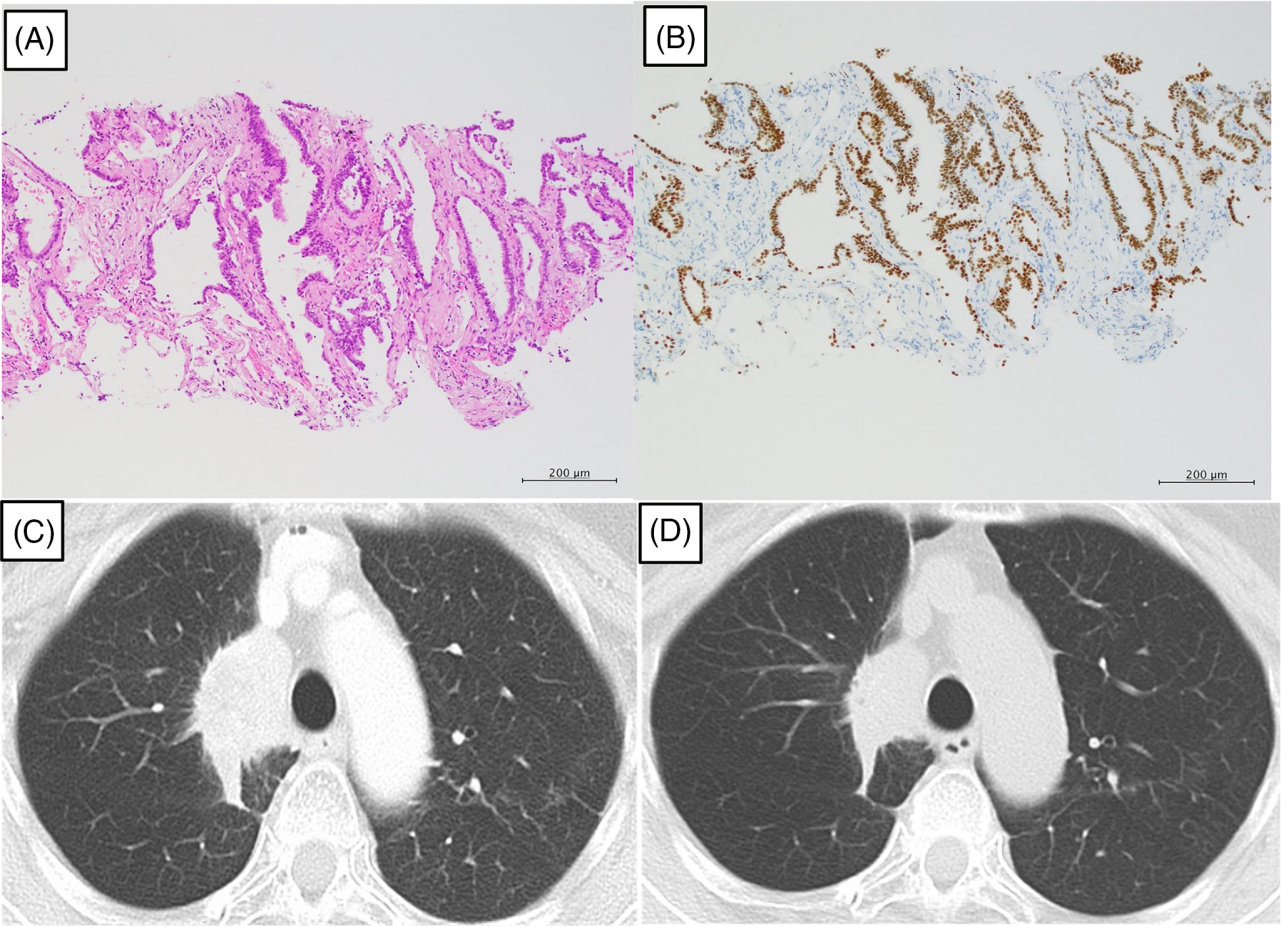
Microscopic histopathology findings. (A) Haematoxylin–eosin staining of the CT‐guided lung biopsy reveals irregularly shaped cells with enlarged, chromatin‐rich nuclei, forming irregularly shaped lumens, partially destroying the existing alveolar structure, and proliferating invasively (×200). (B) Immunostaining reveals infiltration of thyroid transcription factor‐1 positive irregularly shaped cells (×200). (C) CT images before the sixth treatment reveals a mass lesion on the central side of the right upper lobe of the lung. (D) CT images 6 months after olaparib administration reveal a reduction in the primary lesion size.

The chest radiograph taken before the start of the sixth treatment 2 years ago revealed infiltrative shadows in the upper right lung field and hilum, and an increase in the size of these shadows was noted at the time of admission (Figure 1A,B). Chest CT also revealed a growing primary lesion in the right upper lobe. A CT‐guided lung biopsy was performed for the cancer genomic profiling test. The biopsy specimen showed irregularly shaped cells with enlarged, chromatin‐rich nuclei, forming irregularly shaped lumens, and proliferating invasively. Some of these atypical cells were positive for periodic acid Schiff stain, indicating findings consistent with adenocarcinoma (Figure 2A). To exclude lung metastases from breast cancer, staining was additionally performed. The findings were positive for thyroid transcription factor‐1 and napsin A but negative for gross cystic disease fluid protein‐15, which is typically positive in breast cancer (Figure 2B). Thus, the tumour was reconfirmed to be primary lung cancer. Upon submission of this specimen to the cancer genomic profiling test (FoundationOne® CDx Cancer Genomic Profile), a BRCA2 c.9117G > A gene mutation was identified as positive. The variant allele fraction (VAF) was 59.6% (Clin var: Pathogenic), so this mutation indicated that germline mutations were highly likely. We referred the patients and their families to genetic counselling. Her blood test using BRACAnalysis CDx. was also searched for the germline BRCA2 mutation and found positive.

Olaparib, a PARP inhibitor, was selected as the eighth‐line treatment, as recommended by the cancer genomic profiling test and approved by our off‐label committee, following the detection of the BRCA2 gene mutation. It was started at a dose of 600 mg/day, but due to the development of grade 3 anorexia, an antiemetic was used concurrently. Despite this, the symptoms were severe, so the dose was temporarily reduced to 300 mg/day, resulting in improvement. The dose was subsequently increased to 500 mg/day, and the symptoms remained manageable. CEA levels gradually decreased to a minimum of 78.6 ng/mL after starting olaparib, and tumour size was confirmed to have shrunk. Progressive disease (PD) was confirmed 1 year and 9 months after olaparib was started for elevated CEA levels, an increased primary lesion, and a new brain lesion.

Breast cancer was diagnosed as pT1N0M0 stage I after a partial mastectomy 6 months before the introduction of olaparib due to the growth of the lesion. No testing for BRCA2 mutation was performed using tissue of breast cancer.

## DISCUSSION

We report a case of BRCA2‐positive lung adenocarcinoma treated with olaparib as the eighth‐line therapy, resulting in stable disease maintained for about 2 years.

Reports indicate that only 0.79% of patients with primary lung cancer have BRCA2 gene mutations,^1^ and among patients with advanced non‐small cell lung cancer who are negative for EGFR mutation and ALK rearrangement, those with germline BRCA2 mutations account for only 0.5%.^2^ However, a report from China showed that 1.03% (64/6220) of NSCLC patients had pathogenic germline BRCA mutations, and BRCA2 mutations were the most prevalent (49/64, 76.5%).^3^

Tumour‐only analyses such as FoundationOne® CDx Cancer Genomic Profiling cannot determine whether a variant is of germline origin, even with a VAF greater than 50%. Germline BRCA2 gene mutations are inherited from parents to offspring with a 50% probability. In this case, the patient was referred for genetic counselling because her daughter had a history of breast cancer. The possibility that a relative will develop a BRCA2‐related cancer in the future should be carefully explained to the patient and his or her family.

A study has reported a higher frequency of BRCA2 gene mutations in female ex‐smokers with primary lung adenocarcinoma,^4^ which is consistent with the present case. The same study reported a median overall survival of 17.8 months and a progression‐free survival of 9.7 months. In this case, stable disease of primary lung cancer was maintained for approximately 2 years after identifying the BRCA2 gene mutation and olaparib resulted in a progression‐free survival longer than reported in the study. Although treatment for lung cancer patients with BRCA2 gene mutations is being investigated, there is still limited evidence regarding treatment efficacy.

Further research is necessary to elucidate the clinical significance of BRCA gene mutations in primary lung cancer. A cancer genomic profiling test is crucial for selecting appropriate treatment for patients with lung cancer with rare genetic mutations, including the BRCA2 gene mutation, which may lead to an improved prognosis.

## AUTHOR CONTRIBUTIONS

**Takumi Motohashi**: Writing‐original draft; data curation. **Kazutoshi Isobe**: Project administration; conceptualization. **Takahiro Yoshizawa**: Writing‐review & editing. **Yusuke Usui**: Writing‐Review & Editing. **Hiroshige Shimizu**: Writing‐review & editing. **Muneyuki Sekiya**: Writing‐review & editing. **Shion Miyoshi**: Writing‐review & editing. **Yasuhiko Nakamura**: Writing‐review & editing. **Naohisa Urabe**: Writing‐review & editing. **Susumu Sakamoto**: Writing‐review & editing. **Sakae Homma**: Writing‐review & editing. **Sota Sadamoto**: Writing‐review & editing. **Naobumi Tochigi**: Writing‐review & editing. **Kazuma Kishi**: Supervision.

## CONFLICT OF INTEREST STATEMENT

None declared.

## ETHICS STATEMENT

The authors declare that appropriate written informed consent was obtained for the publication of this manuscript and accompanying images. Informed consent was obtained from the patient.

## Data Availability

The data that support the findings of this study are available on request from the corresponding author. The data are not publicly available due to privacy or ethical restrictions.
